# Future developments in sociology in the age of the metaverse

**DOI:** 10.3389/fsoc.2023.1156338

**Published:** 2023-06-22

**Authors:** Vincenzo Auriemma

**Affiliations:** Department of Political and Social Studies, University of Salerno, Fisciano, Italy

**Keywords:** metaverse, sociology, methodological analysis, applied sociology, laboratory research

## Abstract

The intent of this article is to shed light on the future challenges that sociology, in concert with other disciplines, will have to face from now on, starting with one of the possible hypotheses of research methodology. Indeed, as much as some of these issues in the last two decades have become the preserve of neuroscience, their origins, namely the conceptualizations of the classics of sociology, should not be forgotten. In this regard, it will be the task of researchers and sociology to investigate, through applied research attempts, phenomena such as empathy and emotions from innovative methodologies and research different from the current ones, capable of highlighting how emotions are modified by cultural context and spaces of interaction, moving away from a depersonalizing structuralism we were used to, refuting, for example, what neuroscientists have pointed out, according to which empathy and emotions belong to biological universalisms. Therefore, within this brief and informative article we intend to present a possibility of investigation, without any claim to truth or the only way to be able to do research in this field, moved only by the desire to open a fruitful discourse that can lead to the methodological approach toward applied sociology or laboratory research. The idea is to move beyond online netnography, not because the latter cannot provide satisfactory results, but because it will be necessary to broaden the range of choices, such as analysis in the metaverse, generating a viable alternative where this type of analysis cannot be pursued.

## 1. Introduction

Before describing the intent of this paper, it is appropriate to start with a few key concepts, such as the analysis that led to thinking about innovative methodologies for analyzing emotions and that have resulted in research that is now coming to an end. In fact, one of the items under consideration is how empathy can be investigated empirically and, more importantly, how it can be investigated in some of the domains in which we are converging as a society, one of which is the metaverse. Among the various reflections proposed by various scholars that are more or less applicable, two emerged, which could result in two possible paths of investigation: the first is the classical one, that is, observational in nature of the participants and applied through the administration of a questionnaire, for example, the Hogan Empathy Scale, to this first phase it would be appropriate to apply an aspect of analysis that is new to sociology, a kind of laboratory sociology, and could be translated into an emotional analysis on projected figures and images, observing the participants and listening to their reactions. However, this option may not be sufficient, because while it is to be considered, for all intents and purposes, an alternative to empathy analysis, breaking down the intensity of empathy into degrees, the latter may not explain or make one fully understand the empathic dynamics that may occur in people. Therefore, a second hypothesis of investigation could be considered, building on the analyses that are done, for example, in neuromarketing. This second hypothesis aims to observe participants as they are dropped into contexts of active participation within virtual worlds. Therefore, using a viewer, it might be possible to record the interaction activities occurring in this setting and analyze them. Of course, presented in this way, without a theoretical basis to support it, this hypothesis could have enormous limitations. Therefore, to generate theoretical soundness, we need to think about how to enter a virtual world, which for convenience we will call the metaverse, that is, starting from an avatar. In this regard, it will be necessary to describe the concept of embodiment, specifying what is meant by it and why it is essential to use it in experimental sociology research. Therefore, within the next section the theoretical background of this concept will be highlighted.

### 1.1. Theoretical background

To explain the theory related to embodiment described at the end of the introductory paragraph and to have an unambiguous notion of this concept, it is necessary to start with an essential question: what is an avatar? Reading various dictionaries, such as culture, game and video game dictionaries, the avatar is described as an image chosen to represent oneself in virtual communities, places of gathering and discussion, or in online games. This definition clarifies two aspects, the first is that it is not a simple transposition of ourselves, but rather a representation of us, and the second is its symbolic power; the avatar, by representing us in other worlds can embody us or, better, allows the user to fully embody himself, making him feel comfortable, in a virtual body. In this regard, it might be interesting to use the theory of embodiment as the theoretical basis of the methodology.

Specifically, the theory through which to reflect in depth is that relating to the embodiment of emotion and the rethinking of corporeality. In recent years, mainly due to the advent of new technologies and the resurgence of the appreciation of the role of the body and its presence in environments, both physical and virtual, the theory of embodiment has slowly become established. It initially emerged as embodiment cognition, or the analysis of cognition based on the study of the embodied mind. This approach, specifically, supports the idea that the mind should be analyzed in the context of its relationships with a physical body. However, especially between 2018 and 2022, a new avenue for this theory is beginning to emerge, namely the grounded role, which can encapsulate sensorimotor systems, the body, and the social environment, thus no longer just physical. The application of this theory could then be useful in analyzing the interaction between the physical body/virutal body (which we might call the metabody) in relation to other metabodies. The first studies, although embryonic and, at least initially, not part of the conceptualization we know today of embodiment, date back to 1992 with Penrose's analysis. Classical cognitivism has for years been a dominant concept in the world of cognitive science since the second half of the 20th century, the only way in which cognitive processes can be explained; major works include Shapiro's 2007 paper and its reinterpretation done in 2011, but also Wilson's 2002 paper and the reinterpretation done together with Foglia in 2011 (Penrose, 1992; Shapiro, 2007, 2011; Wilson and Foglia, 2011). But also the work of Caiani from 2013, or, again, that of Bermúdez from 2014 (Caiani, 2013; Bermúdez, 2014). According to this approach, the mind can be totally disembodied from its physical support because mental activity is simply the set of computational operations made possible by propositional-inferential representations of the external environment (Fodor, 1981; Johnson-Laird, 1990; Neisser, 2014; Turner and Machalek, 2018).

However, since the early 2000s, artificial intelligence has slowly crept into our daily lives as a somewhat futile attempt to reproduce human intelligence with the goal of creating useful machines. But, as Parisi and Sciortino pointed out, reproducing the entire human brain in a computer does not make us understand the brain, because the reason why it produces its behaviors will continue to be hidden (Parisi and Sciortino, 2013). Net of these extremely relevant details, the role of emotions assumes importance. To understand this phenomenon, it is necessary to break out of the classical patterns of emotions and begin to think about the concept of embodiment of emotions within virtual or virtualized bodies. An example that can be given, to clearly understand the importance of this phenomenon, is the recent research on the impact of technological embodiment through virtual reality on the emotions and emotional involvement of potential hosts, conducted by Flavián et al. (2020). The results were extraordinary, in fact the authors showed “that, compared to computers and cell phones, virtual reality devices evoke more positive emotional reactions and higher levels of psychological and behavioral engagement. Furthermore, emotions and psychological engagement mediate the impact of embodied virtual reality devices on behavioral engagement. The results underscore the importance of technological embodiment in providing immersive pre-experiences in hotels, where hotels incorporate virtual reality into their communication strategies” (Flavián et al., 2020, p. 1). Thus, reasoning within this literature could be very interesting to understand, or at least lay the theoretical groundwork for doing so, what happens within a virtual world. Of course, as well as the Internet, the processes of virtualizing bodies have suffered abrupt setbacks, just think of the various attempts such as Second Life or the more recent Meta. But these elements cannot make us think that we will go no further with the processes of virtual embodiment. In fact, what emerges from another 2021 study by Gall et al. is how the illusion of embodiment in a virtual body provides the means for the modulation of emotional responses. Emphasizing how participants were able to experience more intense arousal, dominance and valence in the high embodiment condition than in the low embodiment condition.

The illusion of embodiment thus intensifies the emotional processing of the virtual environment. This result suggests that artificial bodies may increase the effectiveness of immersive entertainment applications, computer-mediated social interactions, or health applications (Gall et al., 2021). In fact, it is possible to think, in accordance with the 2009 work of Niedenthal, Winkielman, Mondillon, and Vermeulen, that “theories of embodied cognition hold that higher cognitive processes operate on perceptual symbols and that the use of concepts involves a partial reactivation of sensory-motor states that occur during experience with the world. According to this view, emotion knowledge processing involves a (partial) re-experiencing of an emotion, but only when access to the sensory basis of emotion knowledge is required” (Niedenthal et al., 2009, p. 1120).

Therefore, we could reflect on microsociological, everyday-life (I feel like saying) issues of these elements from phenomenology. The idea of using this theoretical aspect arose from an interesting international conference organized by the IRNSN network born within the University of Salerno, particularly following a paper by Professor Paolo Granata. Granata, in agreement with the University of Toronto, in February 2021, at the height of the pandemic, sent VR devices to students in his course to start an experiment. The latter consisted of giving real virtual lectures, but not in DAD as we are used to. It involved wearing a VR helmet and immersing themselves, via a platform, in a meteverse, within which part of the University of Toronto, with attached classrooms and meeting spaces, had been recreated to deliver real lectures. The development of the campus and internal facilities, such as lecterns and classrooms, was done precisely with Unity software. The lecture was a huge success, as evidenced by the video presented on YouTube and available at this link: https://www.youtube.com/watch?v=aRFr46EpHBc. These, as reported by Paolo Granato's Twitter profile, were followed by others: the latest “experiment” was in May 2022, when a workshop was held for high school educators to explore virtual reality and its pedagogical potential (Granato, 2021).

Therefore, it is interesting to see how it is possible to create such a research environment and do applied research in this field of inquiry. One of the ideas that the applied sociology group at the University of Salerno is working on is to virtualize the field of inquiry, specifically applied sociology research, of a laboratory within which to analyze the interactions that will take place in the metaverse in the form of avatars. So, the sample under study will consist of virtualized people, the avatars. Therefore, reasoning within this literature could be very interesting in order to understand, or at least understand what are the theoretical foundations on which to base a methodological analysis suitable for analyzing virtualized and avatar-mediated emotions and relationships.

### 1.2. A methodological research hypothesis

What has been described lays the theoretical foundation but does not analyze a possible methodology. In fact, returning to the main objective of this analysis, it will be useful to reflect on a possible methodology. As anticipated in the previous section, there are several ways in which empathy could be analyzed within applied sociology research, however, only two could provide generalizable results. Assuming that our goal is to understand the extent to which the application of a laboratory-based, experimental method might be useful in investigating the emotions that underlie social interactions and, consequently, understanding whether cognitivists' levels of empathy might be sufficient to analyze all its nuances. One could use the approaches arising out of the dialogue between social neuroscience and sociology, better understood as neurosociology, voluntarily excluding the approach of neuroscience based only on biological constructs, the latter of which make no contribution to emotional analysis from a social point of view (Turner, 2020). However, due to matters of space it is not possible to analyze key works individually, some of which have provided the impetus for the methodological hypothesis described below, out of all of them it is by no means possible to forget or exclude the analyses present in the works of Singer and Lamm, especially that of 2009, which initiated knowledge toward a concept significantly different from the biological one, namely that of the social aspect in neuroscience, such analysis does not make biology their only avenue but consider biological data elements to be integrated with social ones (Singer and Lamm, 2009). Similarly, the work of Brown and Brüne in 2012, or that of Turner, should not be forgotten (Brown and Brüne, 2012). All of these authors, although we do not have space to be able to analyze them individually, have made important contributions to what later came to be called neurosociology and, most importantly, agree that biology alone cannot explain the motivations for people to feel emotions, but only the biological predispositions for a person to feel emotions. Only the interaction of these two elements leads to a clear understanding. Therefore, one could hypothesize the administration of a test that could confirm or refute the collected data identified in the Implicit Association Test, and through a meta-analysis one would try to understand how much social relationships are founded on an empathic basis. The hypothesis of this research work is based on recent findings that have used immersive virtual reality (VR) to analyze violent behavior and the study of anger (Smeijers and Koole, 2019). The approaches described below have been hypothesized for a pilot study, which could aim to understand emotions and the ability to collaborate within teams within a virtual environment, in this case in a metaverse. Therefore, the numbers present, such as sample size or ages, are to be considered purely indicative and suitable, precisely, for a pilot study that aims to understand the data collection power of a research methodology within an experimental sociology context. Given this necessary premise, at this point it is useful to detail our methodology, which will be qualitative-quantitative, and the research path will necessarily have to take place in at least 3 phases.

1. In the first phase, the construction of virtual scenarios will be initiated within which everyday settings will be created that can stimulate interactions among the people under investigation. In addition, images of situations that arouse emotions will be inserted, to analyze the reaction induced in people. The hypothesis we will pursue below will be to use both a virtual environment, accessible through a virtual reality visor, and the setting of a digital environment. Therefore, in our hypothesis and within the same phase, the second piece of hardware, eye-tracking, will be technically set up; its use will be from the same images inserted in the metaverse but projected onto a screen mediated by a projector and a cloth, in this way it will be possible to record the pupil movements of the people taking part in the research, providing details of what elements they tend to dwell on when they experience an emotion and, most importantly, how long they dwell their gaze on those elements. As minor and superfluous as they may seem, these details could lead to a deepening of our investigation, especially considering that empathy not being properly a measurable emotion, could become so through secondary tools that help the researcher.
At this point, an additional concept comes into play, namely embodiment, explored in the previous section. So, participants will experience the perceptual illusion of ownership over a life-size virtual body, embodiment in fact, which visually replaces their own body. Contrary to what one might think, although these virtual bodies may appear drastically different from the participants' own in terms of size, height, skin tone or age, the sampled people will be able to experience a strong subjective feeling of ownership through the personalization of the avatars, as evidenced by embodiment theory. In addition, current technology and, especially, VR scenarios, allow valid experimental setups of the surrounding environment as well, while maintaining a high degree of control (e.g., recreating one's room, a childhood setting, a square or a pub); in fact, individuals will have the illusion of being in a real environment, consequently assuming real but transposed behaviors. Therefore, virtual reality provides a valuable tool for the simulation and study of empathy without exposing participants to any real danger, thus overcoming the ethical problems that arise in non-virtual experiments. What has been emphasized has been previously demonstrated in studies in which virtual reality has been used to assess violent behavior on others, such as Lobbestael and Cima's (2021) study. This phase will allow the generation of an optimal virtual environment capable of performing, without bias, the second phase of research.
2. The second phase will be characterized by sampling and applied research; therefore, non-probability sampling will be used, and specifically, reasoned choice sampling will be used. This is because, not everyone will be able to be sampled, specifically certain subjects will be included. The hypothesis is to perform this research within the sociology undergraduate course, to maintain a strong relevance to both the subject of investigation and to use it as a practical activity during the courses. Thus, the hypothesis could be that the sample will consist of 30 students (15 males and 15 females), this will ensure both to use this study as a pilot study, to limit the physical and economic energies to be expended to reach the first results, and to understand whether and how large the margin of error present in the results will be.
As for the selection of the sample, it will be done according to the following criteria: 20 people will be selected from those between the ages of 19 and 25, who upon administration of the Empathy quotient (EQ), will have achieved a high and average EQ score and 10 people, from the same age group, who will have achieved a low EQ score. In addition, within the same phase, an in-depth, open-ended interview will be conducted, in which case it may be possible to use Charmaz's Grounded Theory methodology, rather than conducting a qualitative analysis based on a rich will be essential to delve into those items of the EQ aimed at analyzing interaction with others, deepening and helping the researcher to understand portions of the lived experience of the people in the sample. At this point, the 30 participants, are to be divided into three equally distributed groups, and referred to as Group I, Group II and Group III; Group I will consist of 10 people who scored high on the EQ and will use only VRs; Group II, similarly to Group I, will consist of 10 people who scored medium on the EQ, but unlike the former, will only use eye tracking; and finally, Group III, which will serve as the control group, will consist of 10 people who scored low on the EQ and will use both VRs and eye tracking; the data collected from the latter will be used as a control on the first two groups. So, a clear distinction also of the tool to be used, the former will be dropped into a virtual environment, the latter into a digital environment, and the third will experience the analysis on both environments. This is because, the starting hypothesis is that introverted people score high on the EQ and that their level of empathy is emotional. In contrast, sensitive and lonely people will achieve medium and low EQ scores, which will fall within the cognitive and compassionate levels, respectively.
In addition, the experimental laboratory analysis will consist of two independent blocks presented in a counterbalanced order; in a first block, emotional images expressing fear and happiness will be displayed, and in a second block there will be expressions eliciting fear and anger. Participants in Group I will wear helmets to observe the two blocks of scenarios at regulated intervals, all for a total duration of 15 min, this is because the virtual reality visor may bring nausea. Participants in Group II will use eye-tracking goggles for a maximum duration of 10 min, as prolonged use of this tool could generate dizziness in subjects, who will be shown the same previous images again but projected. Finally, Group III, will have a total duration, between VR and eye tracking, of 25 min, always maintaining regular intervals between images and, most importantly, a break period of at least 20 min between the use of one piece of hardware and the other. This will allow the researcher to understand how empathy manifests itself in people's lives and in their interactions, as well as allow them to understand whether the levels of empathy highlighted may be sufficient or more will need to be used to bring in the many, many nuances that exist. However, this is not the only methodology that can be used; one could, for example, rotate the three groups so that for each group there is a double analysis. Of course, the process will be more time-consuming, but perhaps it could bring out interesting discordances between using a virtual reality and using a digital reality. Or, again, bring out fundamental confirmations about people's ability to feel empathy within any kind of relationship. All that remains at this point is to apply the third and final phase of the investigation covered by the pilot study.
3. The third and final phase will be characterized by the administration of a questionnaire with the purpose of confirming or not confirming the data collected through the hardware and with the aim of containing the error and exposure of the results. After the experimental phase to all participants, a test will be administered, namely the closing test, the Implicit Association Test; this test has been previously validated and is currently used in the field of social neuroscience, which allows to objectively investigate the strength of association between an item A and an item B, through what are called implicit associations. Such a test will be able to provide an indirect measurement of the strength of association between concepts represented in the images previously displayed within the virtual environment and through eye tracking. This will provide insight into the analysis of the current state and possible future developments.

The second hypothesis of methodology could be related to the Grounded Theory approach. In this case it is possible to proceed with a different application. Much more like earlier research that I personally followed, in collaboration with psychotherapists, based on drop-out in psychology. However, this path although more straightforward to pursue, will be much longer. In fact, it will be necessary to initiate a series of steps preparatory to the search for results. First, once the sample, which could be the same one identified within this paragraph, has been identified, an audio and video recording will need to be made of everything that takes place both within the virtual laboratory and the digital laboratory. Therefore, it will be necessary to encode each recording, and thereafter, it will be essential to investigate all aspects in depth by analyzing them minutely. To do this, it will be necessary to transcribe the recordings, specifying recording code, duration, and mode of administration (VR, eye-tracking or both). This work, hypothetically, could take even more than a year of work, in fact periodic briefings with the entire research team will be necessary to be able to detect any errors in time. However, at the conclusion of the transcription phase of the recordings, we will need to move on to even more important work, namely the selection of the core categories, i.e., the categories of analysis of the phenomenon we want to investigate. Having identified the categories, it will be necessary to create a legend subdividing them by color; this will be necessary so that the analysis of the transcripts can be easily readable and, above all, identifiable. In fact, the categories will have to be identified through further analytical reading of the transcripts. At the conclusion of this step, it will be necessary to census the frequent actions and words within each recording; to facilitate this task, it might be useful to use the NVivo software. Once the frequent words and actions have been identified, it will be necessary to move on to the analysis of each of them.

Obviously, performing such a search using Grounded Theory alone could lead to enormous difficulties along the course of the analysis. In fact, should an error arise, one would have to start the entire search all over again. This might appear to be a major limitation, but it is crucial so that the error can be minimized.

## 2. Conclusion

Without sounding redundant and certain that such an article would have needed more elaboration, both on the proposed methodologies and the authors and theories highlighted, unfortunately this could not be done due to the type of article, namely “Hypothesis and Theory.” However, these methodological hypotheses are not meant to present themselves as the only ones, but only as one of the possible hypotheses that could be applied in this field. Realizing that a lot of funds and, more importantly, a lot of time will be required for its actual implementation, cheaper solutions could be found. One of the hypotheses that have been developed in recent research is to use only eye-tracking as hardware in order to be able to develop the research. The idea originated from a neuromarketing research group in Milan, Italy, which, in order to analyze the power of an advertisement, used this hardware to understand what elements the attention of sample members rested on.

However, we could agree that the speed with which technologies transform our interactions is enormous, so the task of sociology is to be able to read in time the new dynamics of action, which are no longer only physical, but also virtual. For this reason, the researcher will have to open up to new knowledge that often transcends the sociological boundary alone to open up to other sciences, as mentioned earlier and which for matters of space it is not possible to go into here, it might be possible from Turner's concept to carry out pilot studies based on transdisciplinary knowledge that are able to embrace social neuroscience and neurosociology, as well as that of using eye tracking analyses already used within neuromarketing research, such as those carried out by the Neuromarketing Behavior and Brain Lab Research Center at the University of Milan. Of course, the talk just given is just an attempt to present the new challenges that society poses to sociology, trying to analyze interactions that besides being symbolic are mediated by hardware. Perhaps this path will lead to the same results as the classics, however, it is necessary to investigate the power, probably enormous, that these relationships can have in social life.

## Data availability statement

The original contributions presented in the study are included in the article/supplementary material, further inquiries can be directed to the corresponding author.

## Author contributions

The author confirms being the sole contributor of this work and has approved it for publication.
